# The Gut Microbiomes of Two *Pachysoma* MacLeay Desert Dung Beetle Species (Coleoptera: Scarabaeidae: Scarabaeinae) Feeding on Different Diets

**DOI:** 10.1371/journal.pone.0161118

**Published:** 2016-08-17

**Authors:** Philippa Z. N. Franzini, Jean-Baptiste Ramond, Clarke H. Scholtz, Catherine L. Sole, Sandra Ronca, Don A. Cowan

**Affiliations:** 1 Centre for Microbial Ecology and Genomics, Genomic Research Institute, Department of Genetics, University of Pretoria, Pretoria, South Africa; 2 Scarab Research Group, Department of Zoology and Entomology, University of Pretoria, Pretoria, South Africa; Agriculture and Agri-Food Canada, CANADA

## Abstract

Micro-organisms inhabiting animal guts benefit from a protected and nutrient-rich environment while assisting the host with digestion and nutrition. In this study we compare, for the first time, the bacterial and fungal gut communities of two species of the small desert dung beetle genus *Pachysoma* feeding on different diets: the detritivorous *P*. *endroedyi* and the dry-dung-feeding *P*. *striatum*. Whole-gut microbial communities from 5 individuals of each species were assessed using 454 pyrosequencing of the bacterial 16S rRNA gene and fungal ITS gene regions. The two bacterial communities were significantly different, with only 3.7% of operational taxonomic units shared, and displayed intra-specific variation. The number of bacterial phyla present within the guts of *P*. *endroedyi* and *P*. *striatum* individuals ranged from 6–11 and 4–7, respectively. Fungal phylotypes could only be detected within the gut of *P*. *striatum*. Although the role of host phylogeny in *Pachysoma* microbiome assembly remains unknown, evidence presented in this study suggests that host diet may be a deterministic factor.

## Introduction

The microbial gut communities of a wide range of insect species have been investigated (for reviews see [1–6]). The gut environment is considered to be an unstable system, as microorganisms face secretion of digestive enzymes, physical disturbance, habitat shedding during insect moults and other physiochemical conditions that are typically unfavourable for colonisation [1, 2, 6]. However, there are significant benefits to gut colonisation, including high nutrient availability and protection from external environmental stressors [2, 7].

The relationships between host and gut microbiota range across the full spectrum of interactions; i.e., from pathogenic to obligate mutualism [1]. When beneficial to their host, insect-associated microbial communities may participate in a number of activities including degradation of recalcitrant materials such as lignocellulose [8–12], the production of nutrients and vitamins [2, 8, 12], the production of components of cohesion pheromones [13], nitrogen fixation and utilisation of nitrogenous waste products [2, 8, 12, 14], protection against parasites [2, 15], change in body colouration [16] and sterol synthesis [8, 12].

Insect gut microbiomes are known to differ between insect species, driven by variations in the gut structure, different host lifecycles, host phylogeny and diet [2, 6, 17]. The gut microbiome is also influenced within the individual insect or species, varying according to host life-stage [18–22], and/or diet [21, 23–26]. Host diet influences gut microbial communities as they adapt to dietary changes through the induction of enzymes and changes in community structure [23, 27, 28]. However, a core community may persist through major dietary changes [24, 29].

**Fig 1:**
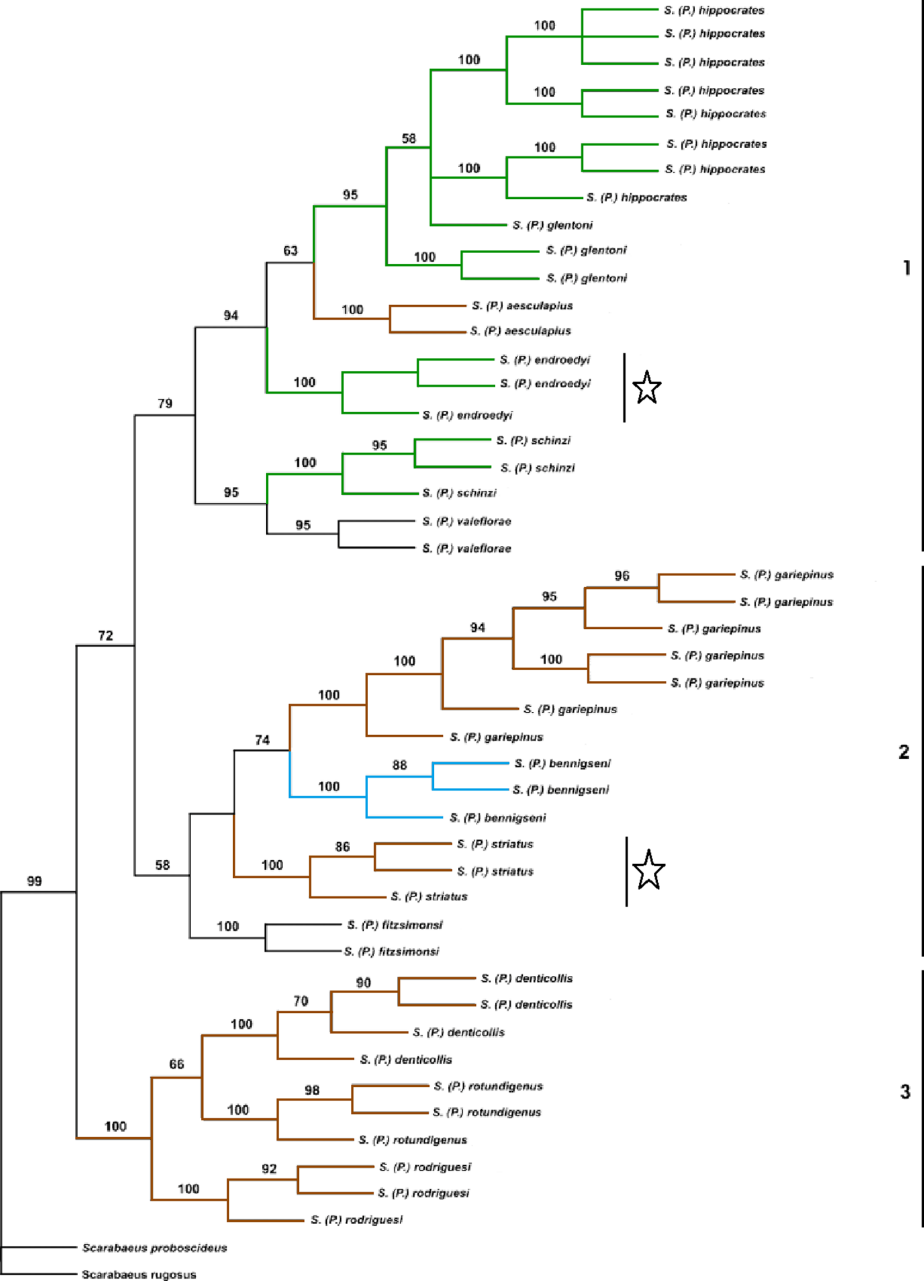
Cytochrome oxidase I gene Parsimony tree phylogeny of 13 *Pachysoma* spp. Branch colours indicate the diet of the *Pachysoma* spp.: dung (brown), plant detritus (green), polyphagous (blue) and unknown (no colour). This phylogentic tree was adapted, with permission, from [42] and the dietary information taken from both [43] and personal observations by Prof C. Scholtz. Two species of *Scarabaeus*, (*S*. *proboscideus* and *S*. *rugosus*) which is the sister-genus to *Pachysoma* and a typical wet-dung-feeder, were used as outgroups. Numbers to the right of the tree indicate the three *Pachysoma* lineages. The two species considered in this study, *P*. *endroedyi* and *P*. *striatum*, are indicated with stars (Adapted from C. Sole [42]).

Studies on insect-microbial associations have mainly focused on termites [4, 30–34], but also on agriculturally important species such as honeybees [35, 36], and medically important insects such as mosquitoes [21, 37–40]. Little attention has been given to dung beetles, which are common and abundant insects in virtually all terrestrial environments and which facilitate nutrient cycling and bioturbation [41]. The desert dung beetle genus *Pachysoma* MacLeay, from the Scarabaeini tribe, of which the quintessential scarab genus, *Scarabaeus* is also a member, consists of 13 species endemic to the south-west African coast [42, 43]. Members of *Pachysoma* exhibit atypical feeding behaviour. While most adult dung beetles feed, by filtration, on minute particulate fragments in wet dung [44, 45], adult *Pachysoma* feed on various and varying dry food sources: plant detritus, dung pellets or both. These substrates are collected on the soil surface and masticated with specially-adapted mouthparts (Fig 1; [42, 43, 45, 46]).

Given that insect gut microorganisms are known to be involved in the degradation of recalcitrant materials such as lignocellulosic compounds [2, 6, 8, 9]), it follows that the gut microbiomes of desert insects may play a significant role in carbon-turnover in desert ecosystems. By studying the gut microbiome diversity of *Pachysoma* spp. feeding on different plentiful and readily-available substrates, it is possible to consider the effects of host diet and/or host phylogeny on gut microbiome assembly processes. This study was designed to characterise the gut microbial (bacterial and fungal) assemblages of coprophagous (*P*. *striatum*) [43] and detritivorous (*P*. *endroedyi*; pers. comm. C. Scholtz) members of the same genus from the same location and to potentially determine whether host diet and/or host phylogeny could be deterministic factors in *Pachysoma* gut microbial community assembly.

## Results and Discussion

### The desert beetle genus *Pachysoma*

The distribution of the *Pachysoma* species is restricted to the arid coastal regions of south-western Africa, principally because of the flightless nature of the genus [43]. The genus *Pachysoma* forms three distinct lineages, supporting six (lineage 1), four (lineage 2) and three (lineage 3) species, respectively [42]. *Pachysoma endroedyi* is located in lineage 1 and *P*. *striatum* in lineage 2 (Fig 1). The driving forces behind the formation of these three lineages are currently unknown. However, it has been noted that all members of lineage 3 have a uniform diet (Fig 1) and originate from desert areas with a consistent aridity index [42, 43], whereas both the aridity index of the desert locations from which lineage 1 and 2 members originate, and their diets, fluctuate (Fig 1).

The diet of *P*. *striatum* consists predominantly of the dry dung pellets [43, 46] of various small native mammalian herbivores and sheep. Despite observations from a decade ago stating that *P*. *endroedyi* was a polyphagous feeder [43], numerous and wide-scale recent observations suggest that *P*. *endroedyi* is a detrivore (Prof C. Scholtz, pers. comm.), the classification adopted in this study. *Pachysoma* species have specialised anatomical and physiological features for mastication and digestion of fragments from plant detritus and dry dung [44, 45].

The linkage between host and gut microbiome is believed to be bidirectional, in that gut microorganisms can provide nutritional assistance to the insect host [12, 47] while the host diet influences the gut microbiome assembly [21, 23–26]. However, host phylogeny may also impact gut microbiome composition [17, 48], irrespective of the diet.

### Sequencing outputs and diversity indices of the bacterial 16S rRNA gene and fungal ITS region of the *Pachysoma* gut microbiome

The gut microbiomes of five detritivorous *P*. *endroedyi* and five coprophagous *P*. *striatum* individuals were determined by 16S rRNA gene amplicon sequencing. After removal of chimeras and singletons, 39050 bacterial and 1492 fungal reads remained, with mean read lengths of 238bp and 100bp, respectively. Only 462 bacterial reads were obtained for *P*. *endroedyi* individual 3 (Table 1), which was therefore removed from further analysis. Considerable variation in the number of bacterial sequence reads was noted between individuals, ranging from 1718 to 2817 and 3911 to 10106 for *P*. *endroedyi* and *P*. *striatum*, respectively. However, Good’s coverage (>0.97 for all samples), rarefaction and chao1 diversity indices suggested that the coverage of *Pachysoma* bacterial gut communities (S1A and S1B Fig) were sufficient for a valid comparison between individuals. The fungal ITS gene region could not be amplified in samples from the detritivorous species *P*. *endroedyi*, despite repeated attempts. The absence of fungi in the insect gut has previously been noted for individuals of various insect groups including Neuroptera and Coleoptera (using culture-dependent techniques: [49]). In the fungal ITS sequence datasets for *P*. *striatum*, diversity indices and rarefaction curves showed low coverage for all but *P*. *striatum* individual 2, suggesting that the fungal diversity was generally underestimated (Table 1; S1C Fig).

**Table 1:** Values for sequence reads, Operational Taxonomic Units (OTUs), phyla and diversity indices for bacterial and fungal gut communities of *P*. *endroedyi* and *P*. *striatum* individuals.

	Individual	Number of reads	Number of OTUs	Phyla	Singletons	Chao	Invsimpson	Shannon	Coverage
Bacterial 16S rRNA gene	*P*. *endroedyi* 1	2120	213	10	105	244.61	19.01	3.88	0.97
Bacterial 16S rRNA gene	*P*. *endroedyi* 2	1718	258	11	133	282.34	81.00	4.91	0.97
Bacterial 16S rRNA gene	*P*. *endroedyi* 3	462	97	11	42	112.62	21.47	3.82	0.94
Bacterial 16S rRNA gene	*P*. *endroedyi* 4	2175	271	6	177	287.59	62.44	4.80	0.98
Bacterial 16S rRNA gene	*P*. *endroedyi* 5	2817	317	9	193	335.83	60.13	4.83	0.98
Bacterial 16S rRNA gene	*P*. *striatum* 1	10106	157	4	84	174.53	8.77	2.82	1.00
Bacterial 16S rRNA gene	*P*. *striatum* 2	4901	158	4	87	194.96	16.64	3.38	0.99
Bacterial 16S rRNA gene	*P*. *striatum* 3	4208	140	6	51	183.05	8.36	2.88	0.99
Bacterial 16S rRNA gene	*P*. *striatum* 4	3911	119	4	71	125.84	8.12	2.93	1.00
Bacterial 16S rRNA gene	*P*. *striatum* 5	6620	172	5	100	201.29	13.36	3.32	0.99
Fungal ITS gene region	*P*. *striatum* 1	136	88	1	156	179.50	96.63	4.29	0.55
Fungal ITS gene region	*P*. *striatum* 2	939	202	2	602	222.81	51.93	4.56	0.94
Fungal ITS gene region	*P*. *striatum* 3	199	106	2	223	248.38	88.35	4.40	0.66
Fungal ITS gene region	*P*. *striatum* 4	107	70	2	153	157.50	65.94	4.04	0.53
Fungal ITS gene region	*P*. *striatum* 5	111	63	2	146	134.75	52.18	3.88	0.62

A total of 1009 bacterial and 294 fungal OTUs were detected at an identity threshold of 97% (Table 1). Numbers ranged from 213 to 317 and 119 to 172 in the *P*. *endroedyi* and *P*. *striatum* gut samples, respectively (Table 1). These values are comparable with results obtained for termite and cockroach gut microbiomes [50]. It should be noted that the fungal ITS sequence read lengths were short (only 100bp), which could explain the poor phylogenetic resolution of *P*. *striatum* fungal gut communities [51].

In both *Pachysoma* spp., the number of bacterial 16S rRNA sequence reads was inversely proportional to the number of bacterial OTUs; i.e., *P*. *striatum* gut samples had a higher average number of bacterial reads (5949 ± 2550) but a lower average number of bacterial OTUs (149 ± 20) when compared to *P*. *endroedyi* (2208 ± 455 reads and 265 ± 43 OTUs, respectively). Those data suggest that the gut bacterial communities of *P*. *striatum* are composed of a relatively low number of dominant phylotypes at high abundance [25, 52, 53]. Contrastingly, the *P*. *endroedyi* gut bacterial community may include a higher bacterial diversity [25, 54]. This inverse relationship, and the higher Shannon diversity index of the *P*. *endroedyi* gut bacterial community (4.6 ± 0.5) compared with the *P*. *striatum* gut community (3.1 ± 0.3; Table 1), suggests that competition is greater in the *P*. *striatum* gut than in *P*. *endroedyi*. This difference may be a reflection of the different diets, as insects feeding on simple diets (e.g., the coprophagous diet of *P*. *striatum*) commonly have a lower gut bacterial diversity than those feeding on more complex diets (e.g., the detritivorous diet of *P*. *endroedyi* [17, 48]).

### Interspecific variations in bacterial and fungal *Pachysoma* gut communities

**Fig 2:**
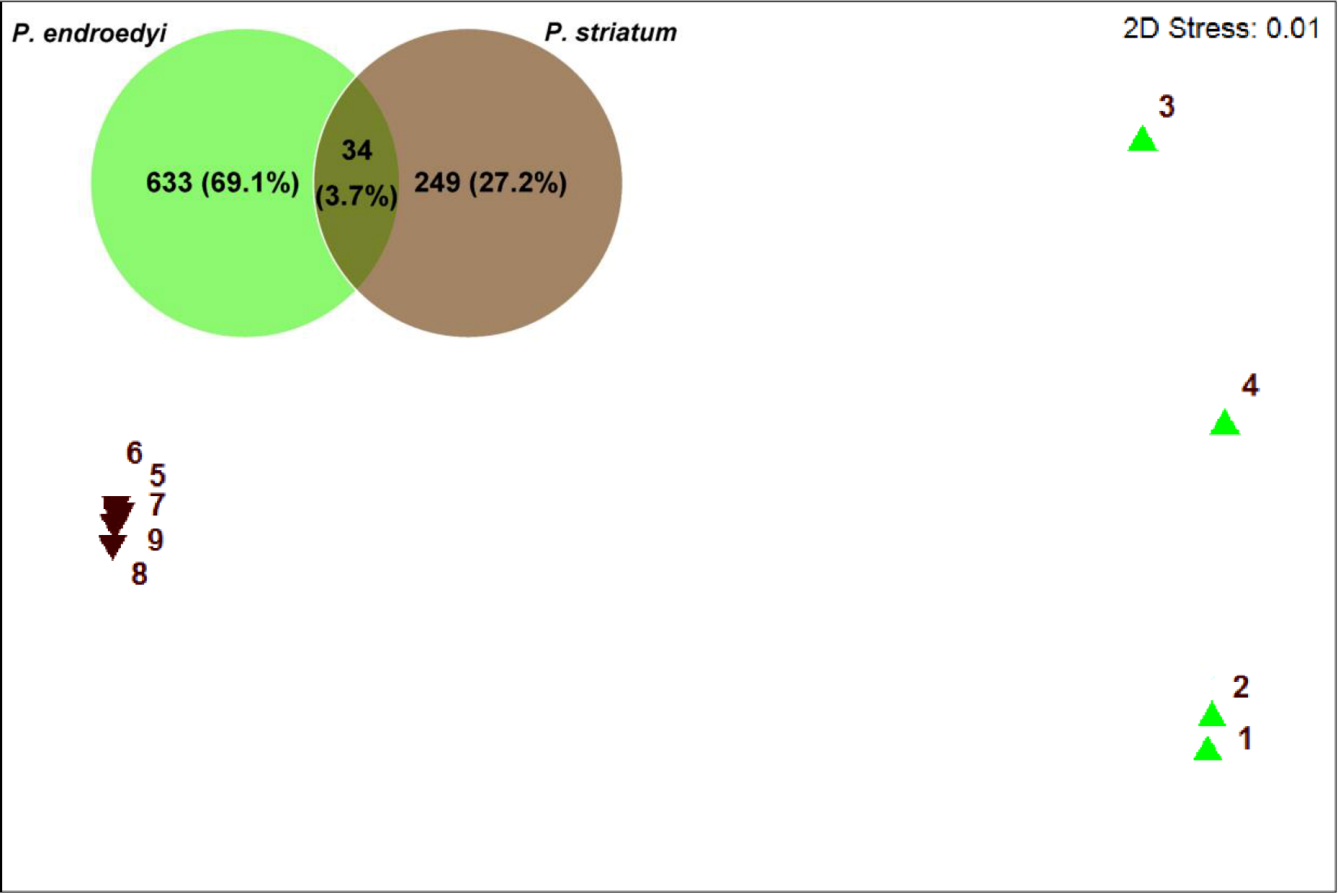
nMDS ordination plot based on Bray-Curtis distance matrices of bacterial 16S rRNA gene pyrosequencing data for *P*. *endroedyi* and *P*. *striatum* individuals. A stress value of less than 0.1 represents a high quality ordination. *Pachysoma endroedyi* and *P*. *striatum* are represented by green and inverted brown triangles, respectively.

The gut bacterial communities of *P*. *endroedyi* and *P*. *striatum* were significantly different, sharing only 3.7% of bacterial OTUs (Fig 2; ANOSIM [R = 1.00, p<0.008]). Both host phylogeny and host diet could be driving forces for the observed differences [17, 55]. For example, the Hymenopteran gut microbiome has previously been shown to be influenced by host phylogeny, while the gut microbiomes of detritivorous insects (e.g., certain termites, Coleoptera and Diptera) are dictated by diet [17]. Gut bacterial communities of *Drosophila* spp. also appear to be impacted by host diet rather than host phylogeny [55]. In Coleoptera (the order in which *Pachysoma* is placed), gut bacterial communities are significantly different to those of other insect groups [17], indicating that host phylogeny is a significant driving force for gut microbiome assembly. However, within Coleoptera, significant similarities in bacterial assemblages of certain beetles with similar diets (e.g., those feeding on live arboreal tissue) have also been noted [17], which suggests that diet may also be a deterministic factor. It should, however, be noted that no coprophagous insects were included in this study [17], making a direct comparison with *Pachysoma* speculative.

It is not possible to compare the gut fungal communities of the two insect species studied, given that despite numerous attempts we were unable to PCR-amplify fungal ITS sequences from the detritivorous *P*. *endroedyi*. While we think it unlikely that fungal species are completely absent from the gut microbiome of this species, this negative result suggests that they may represent a relatively minor fraction of the total gut microbial diversity. To fully confirm this, the sample size should be increased and *P*. *endroedyi* individuals from multiple breeding populations should be investigated.

We would expect host diet to be a contributing factor in the presence (or absence) of fungi in the *Pachysoma* gut. For example, true yeasts (Saccharomycetes) are typically observed in the guts of litter-, plant- and wood-feeding insects [56–58], but not in those of predacious insects [49, 59].

### Intraspecific variation of *Pachysoma* gut microbial communities

**Fig 3:**
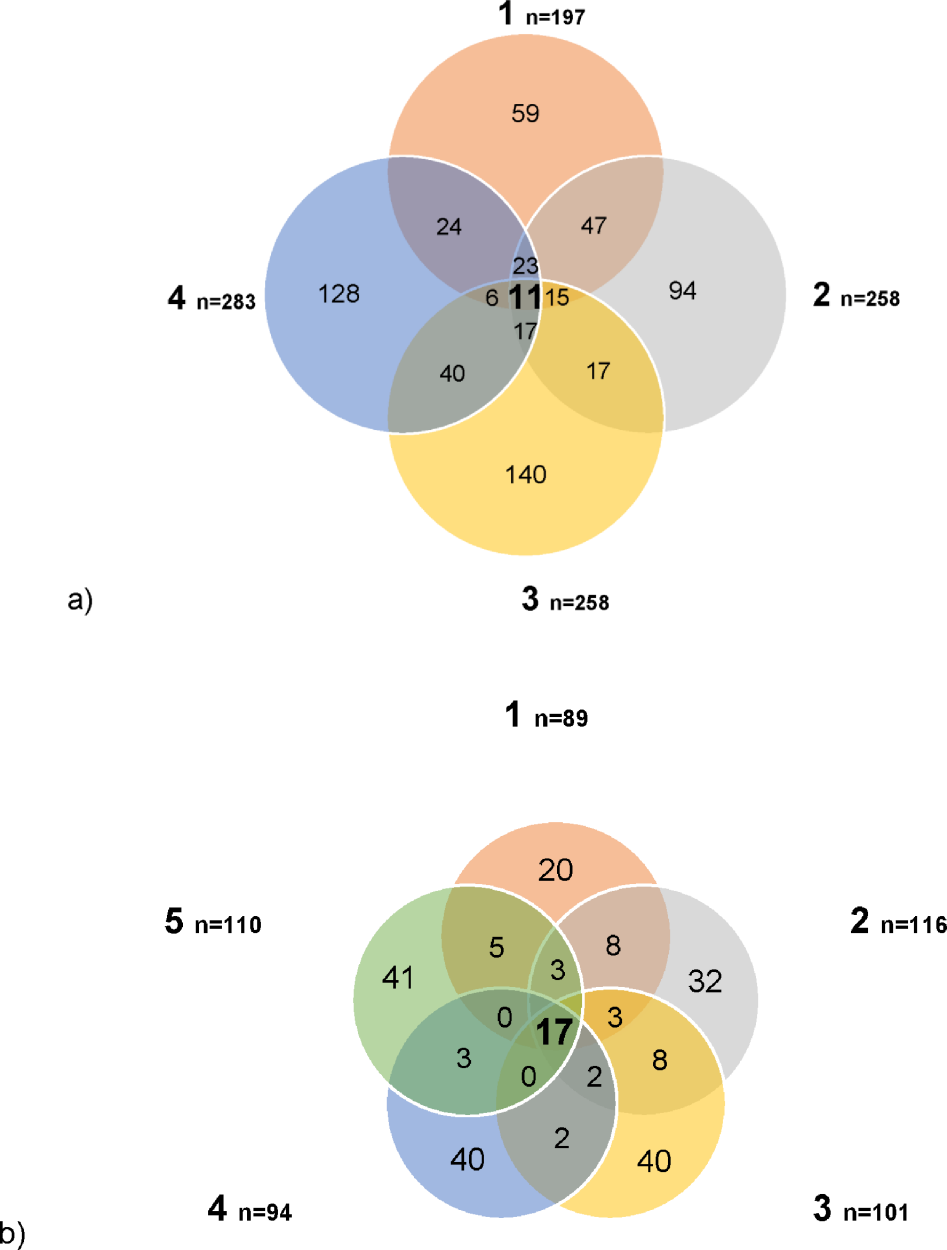
**Venn diagrams showing distribution of bacterial OTUs between (a) *P*. *endroedyi* and (b) *P*. *striatum* individuals based on the 16S rRNA gene pyrosequencing analysis.** Shared OTUs are shown in bold. Numerical labels are shown for each individual.

**Fig 4:**
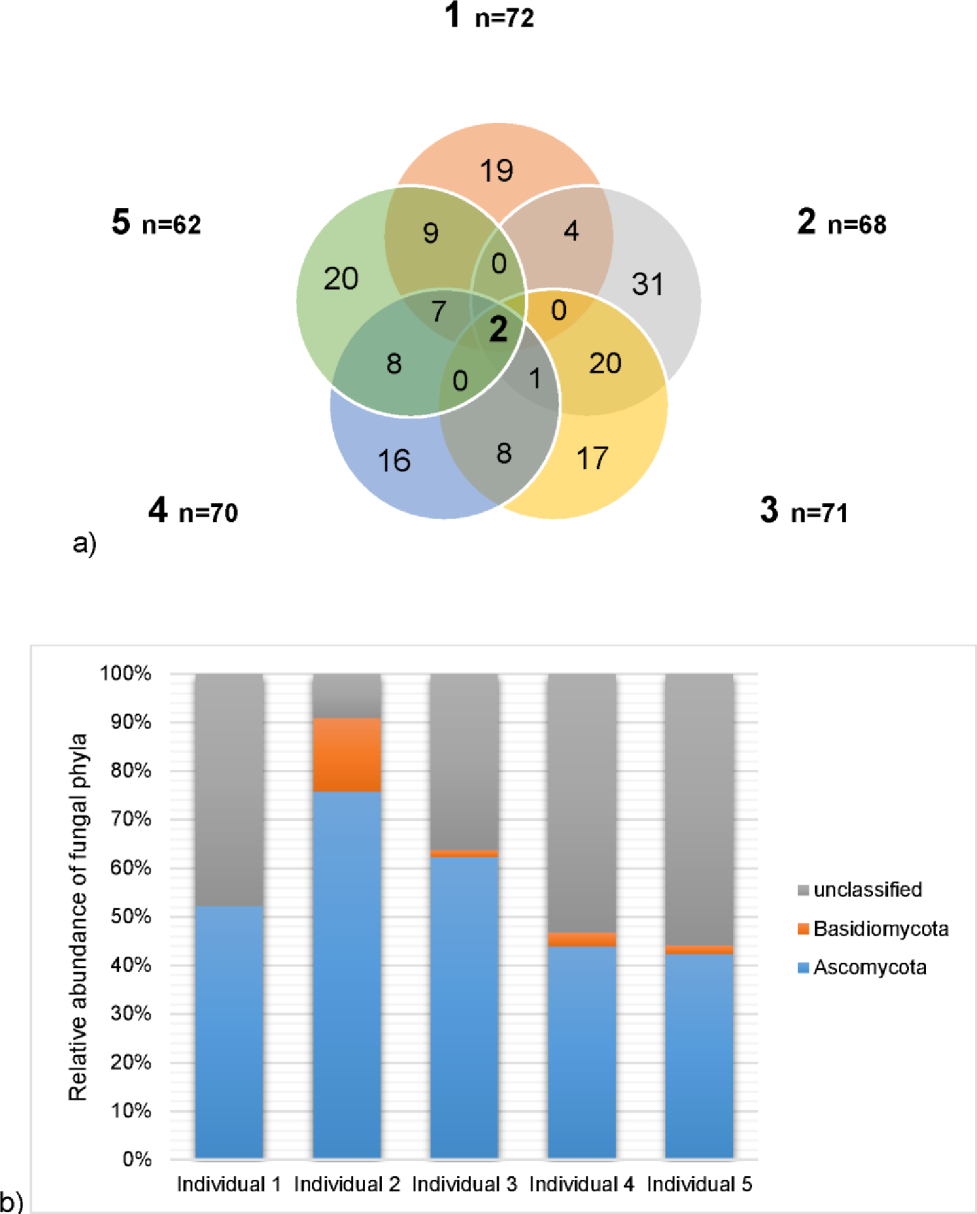
**a) Venn diagram comparing the distribution of fungal OTUs between *P*. *striatum* individuals based on the ITS gene region pyrosequencing analysis and (b) relative abundance of fungal phyla in five *P*. *striatum* individuals based on ITS rRNA gene region pyrosequencing analysis at a 97% identity threshold.** Shared OTUs in the Venn diagram are shown in bold with numerical labels given for each individual.

Large intraspecific differences in *Pachysoma* gut communities were noted, with the majority of OTUs being unique to each *Pachysoma* individual (Fig 3A and 3B, Fig 4A) and only 11 (1.1%) and 17 (3.3%) bacterial OTUs being shared between individuals of *P*. *endroedyi* and *P*. *striatum*, respectively. Furthermore, only two non-abundant fungal OTUs (ranging from 1.6–1.7% of the community) were shared among the five *P*. *striatum* individuals (Fig 4A). Such intraspecific differences, relating to the relative abundances and diversity of bacterial members of gut communities, are not uncommon, as has been observed for honeybees (*Apis cerana* and *A*. *mellifera* [52]), mosquitoes (*Aedes* spp., *Culex* spp., *Anopheles* spp., *Mansonia* spp.; [37, 38]) and the red palm weevils *Rhynchophorus ferrugineus* and *R*. *vulneratus* [25], among others. A recent study on the gut microbiomes of 218 different insect species from 21 orders [48] indicated that 46% of the total number of bacterial OTUs detected (n = 9301) were only observed in single individuals. The large intraspecific variation noted in *Pachysoma* could be influenced by the stochastic, and transient, process of microorganisms entering the gut with the food source [2] and, for *P*. *striatum*, the different amounts of feeding material contained in the guts of each individual [1]. Furthermore, it cannot be excluded that the ‘time of feeding’ prior to sampling may also have had an influence on intraspecific gut microbiome variability [1].

Of the shared bacterial OTUs, only one (assigned to the phylum Bacteroidetes) and eight (4 Firmicutes, 2 Actinobacteria, 1 Bacteroidetes and 1 Proteobacteria) were abundant (i.e., represented >2% reads) in the *P*. *endroedyi* and *P*. *striatum* gut samples, respectively. This distribution is strongly suggestive that the *Pachysoma* gut core community is very small, as has been proposed for the “minimal core” model [60]. Other studies have noted the presence of consistent core microbial communities within individuals of the same insect species (e.g., the bed bug *Cimex lectularius*; [61] and bumble bee *Bombus terrestris* [62, 63]), or across taxonomic levels (e.g., across the ant tribe Cephalotini; [64]). In the termite *Reticulitermes flavipes*, a substantial core bacterial microbiome (65% shared OTUs) was noted, regardless of the artificial feeding diet, suggesting that host phylogeny may play a more important role than host diet in the assembly of the gut microbiome [24]. Similar results have been noted in cockroaches [65]. However, with a minimal core microbiome in both *Pachysoma* spp., phylogeny appears less important than diet. Furthermore, a minimal core gut microbiome may result from negative interactions between gut microorganisms, such as antagonism or amensalism, or indicate, as for Drosophila [66], the establishment of ‘non-gut-specific’ microorganisms.

It has been suggested that a ‘functional’ rather than a ‘phylogenetic’ core microbiome may be more informative in determining the assembly of gut microbiomes [67]. In studies on humans, which typically follow the minimal core model, functional gene diversity appears to be broadly similar across individuals [67, 68]. Therefore, there may be a functional core community in each *Pachysoma* spp. studied, displaying shared metabolic capacities [68]; i.e., exhibiting functional redundancy. As such, it has been suggested that a comparison of functional properties of hosts feeding on different diets can guide an understanding of the functional roles of different gut microbiomes [67].

### Phylogenetic diversity of bacterial and fungal *Pachysoma* gut communities

**Fig 5:**
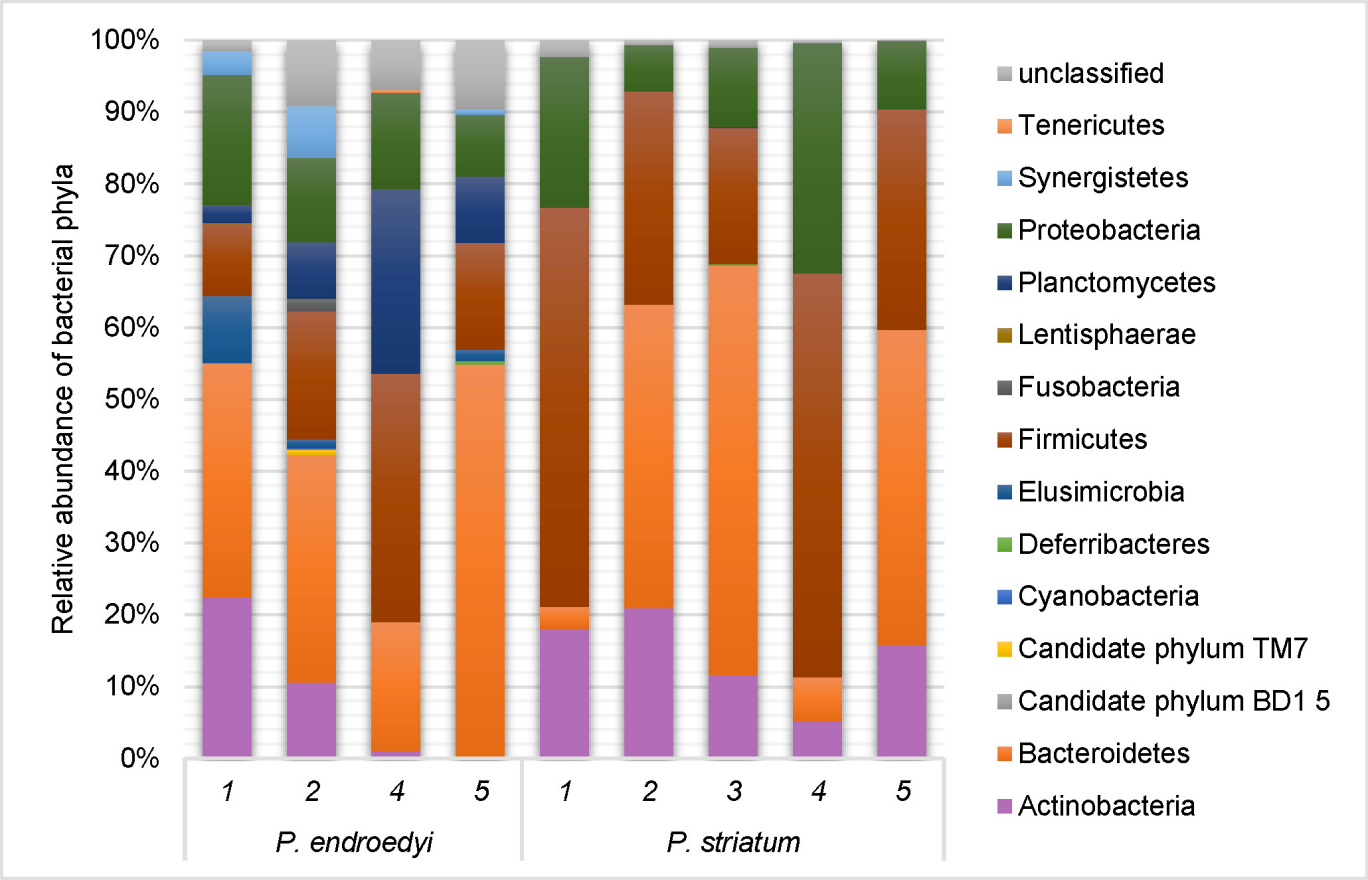
Comparison of interspecific differences in relative abundance of bacterial phyla in the gut of two *Pachysoma* spp., *P*. *endroedyi* and *P*. *striatum*, based on 16S rRNA gene pyrosequencing analysis.

The gut bacterial diversity of *P*. *endroedyi* was higher (6–11 phyla; Fig 5) than that of *P*. *striatum* (4–7 phyla; Fig 5). The *P*. *endroedyi* gut samples were dominated by Bacteroidetes (18.0–54.8%), Firmicutes (10.0–34.6%), Proteobacteria (8.7–18.1%) and Planctomycetes (2.5–25.7%), while Actinobacteria (0.1–22.5%), Elusimocrobia (0–9.3%) and Synergistetes (0–7.3%) showed highly variable abundances (Fig 5). The remaining 7 phyla each represented less than 2% of the community and were often detected in single insects. In *P*. *striatum*, Bacteroidetes (3.0–57.1%), Firmicutes (18.9–56.2%), Proteobacteria (6.4–32.1%) and Actinobacteria (5.2–21.0%) were also dominant phyla although the relative abundances varied between individuals (Fig 5). Three minor phyla (<2% abundance) were only detected in two *P*. *striatum* individuals, namely Deferribacteres, Planctomycetes and Synergistetes (Fig 5). All the identified bacterial phyla have previously been reported in insect microbiomes, with the phyla Firmicutes, Proteobacteria, Bacteroidetes and Actinobacteria commonly abundant in insect gut samples [48, 69].

The presence of specific bacterial phyla and/or their relative abundances in insect gut samples may be linked to host diet. For example, certain insects with simpler diets (e.g., feeding on pollen and nectar [52], fruit [22, 70], or sap [71]), contain gut bacterial communities which are typically dominated by heterotrophic Proteobacteria and/or Firmicutes. In contrast, Bacteroidetes (along with other phyla) were highly abundant in the gut microbiomes of insects feeding on plant materials such as wood and leaves [25, 54, 65, 72, 73]. The *P*. *striatum* gut bacterial communities did not display these patterns, suggesting that coprophagous diets may structure insect gut communities differently.

Fifteen and 11 bacterial genera were abundant (>2% relative abundance of reads) within the guts of *P*. *endroedyi* and *P*. *striatum*, respectively (Table 2). Only two of these genera were abundant in both species (*Dysgonomonas* and unclassified Enterobacteriaceae; Table 2). *Dysgonomonas* was less abundant in *P*. *endroedyi* gut samples (2.8% ± 0) than in *P*. *striatum* (26.3% ± 0.2), in which it was the most abundant genus. *Dysgonomonas* have been reported to be present at high abundance in the gut system of the fungus-growing termite (*Macrotermes annandalei*) and red palm weevil larvae (*Rhynchophorus ferrugineus*) [74, 75]. Two species of *Dysgonomonas* have previously been characterised from the gut of termites [76, 77]. Both species have been found to ferment glucose and xylan as a sole carbon source and to produce acetic acid as the major end-product [76, 77], suggesting roles in both the lignocellulosic biomass degradation pathway and in providing readily metabolisable substrates for ingestion by the host. The large difference in the abundance of this phylotype in the two *Pachysoma* species suggests a key nutritional role in *P*. *striatum* but not in *P*. *endroedyi*.

**Table 2:** Phylogenetic classification of the most abundant bacterial genera in the gut samples of *P*. *endroedyi* and *P*. *striatum*: i.e., representing >2% reads.

Phylum	Family	Genus	*P*. *endroedyi* (%)	*P*. *striatum* (%)
Actinobacteria	Propionibacteriaceae	*Proponiobacterium 1*	8.2 ±0.1	0.1 ± 0
Actinobacteria	Propionibacteriaceae	*Tessaracoccus*	0	7.6 ± 0
Actinobacteria	unclassified	*unclassified*	0.1 ± 0	5.2 ± 0
Bacteroidetes	Bacteroidaceae	*Bacteroides*	7.6 ± 0	0
Bacteroidetes	Marinilabiaceae	*Uncultured 1*	2.7 ± 0	0
**Bacteroidetes**	**Porphyromonadaceae 1**	***Dysgonomonas***	2.8 ± 0	**26.3 ± 0.2**
Bacteroidetes	Porphyromonadaceae 4	*Proteiniphilum*	0.2 ± 0	3.5 ± 0
Bacteroidetes	Rikenellaceae	*Alisitpes IV*	6.5 ± 0	0
Bacteroidetes	Rikenellaceae	*unclassified*	3.2 ± 0	0
Bacteroidetes	unclassified	*unclassified*	5.3 ± 0	0.1 ± 0
Elusimicrobia	Endomicrobiaceae	*Endomicrobium*	3.0 ± 0	0
Firmicutes	Enterococcaceae	*Vagococcus*	0.3 ± 0	4.9 ± 0
Firmicutes	Family XI Incertae Sedis	*unclassified*	0	10.9 ± 0.1
Firmicutes	Lachnospiraceae	*Uncultured 13*	0.9 ± 0	2.9 ± 0
Firmicutes	Lachnospiraceae	*unclassified*	3.3 ± 0	0.7 ± 0
Firmicutes	Ruminococcaceae	*Termite cockroach cluster*	5.3 ± 0.1	0.1 ± 0
Firmicutes	Ruminococcaceae	*unclassified*	3.3 ± 0	1.4 ± 0
Firmicutes	Veillonellaceae	*Anaeroarcus-Anaeromusa*	0	11.5 ± 0.1
**Planctomycetes**	**unclassified**	***unclassified***	**11.3 ± 0.1**	0
Proteobacteria	unclassified	*unclassified*	0	3.1 ± 0
Proteobacteria	Insect cluster	*unclassified*	2.2 ± 0	0
Proteobacteria	Enterobacteriaceae	*unclassified*	4.2 ± 0.1	7.4 ± 0.1
Proteobacteria	Enterobacteriaceae 1	*unclassified*	0	2.5 ± 0.1
Synergistetes	Synergistaceae	*Candidatus Tammella*	2.8 ± 0	0

Percentages are the average read relative abundances in each species (*P*. *endroedyi*: n = 4; *P*. *striatum*: n = 5). Colours depict the species in which the bacterial genus is abundant: *P*. *endroedyi* (green), *P*. *striatum* (brown) or both (blue). The most abundant genus of each species is shown in bold.

An unclassified Planctomycetes dominated the gut samples of *P*. *endroedyi* (11.3% ± 0.1 relative abundance of reads). To the best of our knowledge this is the first report of an insect gut microbiome dominated by Planctomycetes. Planctomycetes were only detected in a single *P*. *striatum* individual at very low abundance (0.01%). Planctomycetes have previously been detected in the guts of the termites *Syntermes wheeleri* and *Nasutitermes* spp. [58, 72], the cockroach *Shelfordella lateralis* [65], adult and larval beetles (*Cryptocephalus* spp., *Prionoplus reticularis* and *Pachnoda* spp.; [78–80]), the tree weta *Hemideina thoracica* [73] and the mosquito *Aedes albopictus* [81], but only in low abundances (<1–5% relative abundance).

Ascomycota was the most abundant fungal phylum (42.3–75.7%) in all *P*. *striatum* gut samples, which is typical for insect gut microbiomes [57, 58, 82, 83]. Basidiomycota were not ubiquitously detected, and were observed only in the gut samples of four of the five *P*. *striatum* individuals (1.8–15.2%; Fig 4B). A substantial proportion of fungal ITS sequence reads could not be classified, even at the phylum level (9.1–55.9%; Fig 4B). Unfortunately, relatively little is known about insect gut fungal diversity (compared to bacterial diversity [84]), with the majority of published studies being based on culture-dependent methods which are typically biased when compared with culture-independent methods [84, 85].

## Conclusion

This is the first study to investigate the gut microbiomes of any dung beetle feeding on dry food sources and to compare those of closely related adult dung beetle species with very different diets but from the same locality. *Pachysoma* spp. are ecologically important in arid environments where they undoubtedly participate in nutrient cycling and bioturbation [41]. We have demonstrated that, as predicted, the gut microbiomes differed significantly between two species which feed on different substrates. However, both populations showed large intraspecific variations. Thus, to further characterise the gut microbiomes of these *Pachysoma* species, the number of individuals studied should be increased and populations from different sites investigated. Such experiments would make it possible to evaluate whether interspecific variation was higher than intraspecific variation within a single *Pachysoma* species.

We are unable to fully assess whether host phylogeny or the host diet is the dominant driver of the *Pachysoma* gut microbiomes. Nevertheless, we provide evidence that diet probably plays a significant role, particularly noting the fact that the gut microbiomes of the detritivorous *P*. *endroedyi* (feeding on complex food sources) have higher bacterial diversities than those of the coprophagous species (feeding on relatively simple food sources) [17, 48]. Functional gene analysis of the microbiomes of *P*. *endroedyi* and *P*. *striatum* could potentially assist in confirming the role that host diet plays in *Pachysoma* gut microbiome assembly [11].

## Experimental procedures

### Collection and storage of *Pachysoma* spp

Five adult individuals of *P*. *endroedyi* and of *P*. *striatum* (S2 Fig) from single breeding populations, feeding on plant detritus and dung respectively, were collected by the Scarab Research Group in September and October 2014 from coastal sandveld near Kommandokraal, Namaqualand, South Africa (S31°29'58.4" E18°12'29.2") under the Cape Nature permit number 0056-AAA008-00041. Ethical clearance is not necessary for work carried out on insects. Beetles were identified at the site. Due to their size, 99% ethanol was injected into their abdomens using sterile syringes for gut preservation [25]. Insects were then stored in 99% ethanol at -80°C, until dissection.

### Gut dissection

Gut dissections were performed under a Zeiss Stemi 2000-C dissection microscope (Zeiss, Oberkochen, Germany) as previously described [86] with minor modifications. All equipment was sterilised before use with 10% bleach and 70% EtOH. The average body length of *P*. *endreodyi* ranges from 20.7–26.4mm, and the one of *P*. *striatum* ~19 mm [43]. The insects were placed in a wax-lined glass Petri dish with quarter strength autoclaved Ringer solution (0.12 g/L CaCl_2_, 0.105 g/L KCl, 0.05 g/L NaHCO_3_, 2.25 g/L NaCl; Sigma-Aldrich). The thorax and abdominal integument were removed using scissors before pinning the specimen to the wax layer in the Petri dish. Forceps were used to remove the membranes covering the internal organs. The rectum was pulled downwards, moving the gut gently out of the body cavity. The five *P*. *endroedyi* guts appeared full of diet material while the *P*. *striatum* ones were empty (n = 1), half-full (n = 1) or full (n = 3). Hindgut and midgut samples were separated and stored in 1.5ml eppendorf tubes at -20°C until DNA extraction.

### Metagenomic DNA extraction

Gut-section metagenomic DNA extractions were performed using a modified version of the protocols previously described by [87, 88]. Whole-guts were weighed and crushed in liquid nitrogen using sterile mortars and pestles. For 10mg of gut, 100μl of a preheated (60°C) 2% CTAB solution (0.1M Tris HCl [pH8.0], 1.4M NaCl, 0.02M EDTA [pH8.0]) was added. The mixtures were incubated for 30min at 60°C before centrifugation for 5min at 10000rpm. The supernatant was transferred to a clean collection tube and enzymatic digestion of the gut samples was carried out with the addition of 2μl lysozyme (5mg/ml) per 100μl CTAB solution for 30min at 37°C under continuous shaking (120 rpm). 0.5μl Proteinase K (20mg/ml) per 100μl CTAB was then added [89], followed by an overnight incubation at 55°C with continuous shaking. One volume phenol:chloroform:isoamyl alcohol (25:24:1) solution was added. Tubes were inverted and centrifuged at 13000rpm at 4°C for 4min. One volume chloroform:isoamyl alcohol (24:1) solution was added to the top aqueous phase and the mixtures were inverted before centrifugation at 13000rpm at 4°C for 15min. This step was repeated until no protein contamination was observed [89]. DNA was precipitated with 3M NH_4_Ac [90] and ice cold 99.9% EtOH followed by overnight incubation at -20°C. Mixtures were centrifuged for 60min at 14000rpm at 4°C. The DNA pellet was washed twice with ice cold 70% EtOH and allowed to dry completely for 2 hours. The DNA pellet was resuspended in 50μl filter-sterilized nanopure H_2_O overnight at 4°C [90], and stored at -20°C for downstream analysis.

### 454 pyrosequencing of the bacterial 16S rRNA gene and fungal ITS gene region

The gut metagenomic DNA of five individuals (equal concentrations of combined hindgut and midgut-derived DNA) from each *Pachysoma* spp. was sent to Molecular Research (www.mrdnalab.com) for 16S rRNA gene and ITS gene region pyrosequencing using the Roche 454 GS FLX titanium platform. The primers 27F (AGRGTTTGATCMTGGCTCAG; [91]) and 338R (AGTGCTGCCTCCCGTAGGAGT; [92] were used to amplify the 16S rRNA gene region as they have a low eukaryotic coverage (27F: 0%; 338R: [93]). Fungal specific fITS9 (GAACGCAGCRAAIIGYGA; [94]) and ITS4 (TCCTCCGCTTATTGATATGC; [95]) primers were used for the amplification of the ITS gene region.

### Data analysis

Raw pyrosequencing reads were filtered and analysed using MOTHUR version 1.35.1 (Accessed May 2015- January 2016; [96, 97]. In short, fasta, quality and flow files were extracted from the sff files using the sff.info command. For the bacterial 16S rRNA gene pyrosequencing reads, filtering of poor quality reads was done using the shhh.flows command allowing for reads to have one or two mismatches between the barcodes and primers respectively. Remaining sequences were quality filtered with the trim.seqs command to maximum homopolymers of 8bp and a minimum sequence length of 100bp. Sequences were aligned to the SILVA reference database (http://www.arbsilva.de/download/arb-files/) using the align.seqs command. The screen.seqs and filter.seqs commands were used to retain only overlapping sequences. Chimeras were identified and removed using the chimera.uchime command. Sequences were classified against five databases, namely the Ribosomal Database Project (RDP), SILVA, NCBI, The Dictyoptera gut microbiota reference Database (DictDb; data shown) and GreenGenes with a confidence threshold of 80%. OTUs were clustered for each individual beetle before removal of singletons using the remove.rare command. Samples were subsampled 1718 reads, i.e., the lowest number of reads across all samples.

ITS reads were analysed similarly to that of the 16S reads with minor differences as outlined previously [98]. Filtering of poor quality reads was done using the trim.seqs command allowing for reads to have one or four mismatches between the barcodes and primers respectively. Sequences were trimmed to 200bp using the chop.seqs command to ensure all sequences were the same length. Sequences were classified against the UNITE database (Version 6) with a confidence threshold of 50% and subsampled to the lowest number of OTUs across all samples (107) for statistical analyses.

Phylogenetic comparisons, of both the bacterial and fungal datasets, were done using the relative abundance of all reads in the dataset so as to ensure inclusion of rare taxa. Relative abundances (%) were calculated from the number of reads of the microbial organism(s) in question divided by the total number of reads for the particular *Pachysoma* individual.

Nucleotide sequences for both the bacterial and fungal datasets have been uploaded to NCBI (http://www.ncbi.nlm.nih.gov/) Short Read Archive (SRA) under the accession number SRP071915.

### Statistical Analysis

Two-dimensional Non-Metric Multi-Dimensional Scaling (nMDS) plots were constructed in Primer 6 software (version 6.1.5.81 (Primer E Ltd, Plymyth, UK)) after applying square-root pre-treatment and using the Bray-Curtis coefficient [99] to build a dissimilarity matrix. Kruskal’s stress value was used to determine the efficiency of sample placement in both two- and three-dimensional nMDS plots. Significant differences in bacterial gut communities were determined using one-way global Analysis of Similarities (ANOSIM) in Primer 6 software version 6.1.5.81 (Primer E Ltd, Plymyth, UK) using 10 000 permutations [100]. A Venn plot was created using R (2.15.1) (www.rproject.org) to differentiate between unique and shared OTUs dependent on feeding strategy. Diversity indices and rarefaction curves were generated in Mothur [97]. Singletons were removed prior to analyses.

## Supporting Information

S1 Fig Rarefactions curves showing gut microbial community richness of all *Pachysoma* individuals for bacterial 16S rRNA gene amplicon data of: a) *P*. *endroedyi*, b) *P*. *striatum*; and c) fungal ITS gene region amplicon data of *P*. *striatum*.
(TIF)

S2 Fig Photographs of *P*. *endroedyi* (a) and *P*. *striatum* (b) in their natural environment before collection (courtesy of Hennie de Klerk).
(TIF)
