# Correction: The Gut Microbiomes of Two *Pachysoma* MacLeay Desert Dung Beetle Species (Coleoptera: Scarabaeidae: Scarabaeinae) Feeding on Different Diets

**DOI:** 10.1371/journal.pone.0165376

**Published:** 2016-10-19

**Authors:** Philippa Z. N. Franzini, Jean-Baptiste Ramond, Clarke H. Scholtz, Catherine L. Sole, Sandra Ronca, Don A. Cowan

The accession number listed in the Data Availability statement for this paper is incorrect. The correct statement is: Sequences for both the bacterial and fungal datasets have been uploaded to NCBI (http://www.ncbi.nlm.nih.gov/) Short Read Archive (SRA) under the accession number SRP071915.
