# Supplementary figures and images for: The Gut Microbiomes of Two Pachysoma MacLeay Desert Dung Beetle Species (Coleoptera: Scarabaeidae: Scarabaeinae) Feeding on Different Diets
Source: PLoS One. 2016 Aug 17;11(8):e0161118. doi: 10.1371/journal.pone.0161118 (PMC4988786; doi:10.1371/journal.pone.0161118)

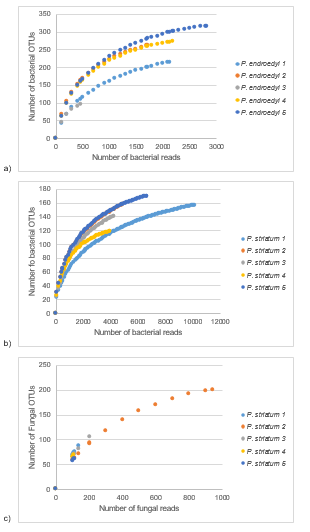

Supplement: S1 Fig — Rarefactions curves showing gut microbial community richness of all Pachysoma individuals for bacterial 16S rRNA gene amplicon data of: a) P. endroedyi, b) P. striatum; and c) fungal ITS gene region amplicon data of P. striatum. (TIF) [file pone.0161118.s001.tif]

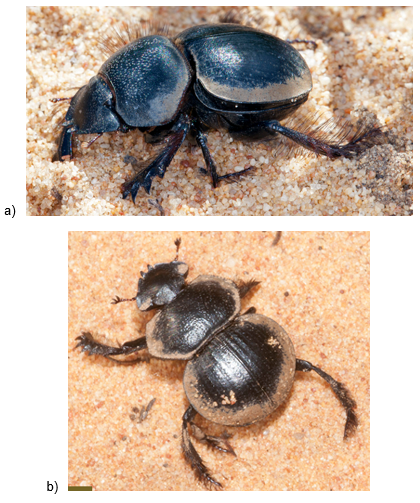

Supplement: S2 Fig — Photographs of P. endroedyi (a) and P. striatum (b) in their natural environment before collection (courtesy of Hennie de Klerk). (TIF) [file pone.0161118.s002.tif]
